# Editorial: Tuberculosis Drug Discovery & Development: Drug Targets, Chemical Matter, and Approaches

**DOI:** 10.3389/fcimb.2021.755459

**Published:** 2021-09-09

**Authors:** Vinayak Singh

**Affiliations:** ^1^Drug Discovery and Development Centre (H3D), University of Cape Town, Rondebosch, South Africa; ^2^South African Medical Research Council Drug Discovery and Development Research Unit, Department of Chemistry and Institute of Infectious Disease and Molecular Medicine, University of Cape Town, Rondebosch, South Africa

**Keywords:** tuberculosis, drug discovery, drug target, drugs, *Mycobacterium tuberculosis*

Tuberculosis (TB), caused by *Mycobacterium tuberculosis* (*Mtb*), is the leading cause of death from a single infectious agent ranking it above all other contagious diseases. About one-third of the world’s population is carrying *Mtb* and are at high risk of developing active TB, signifying the severity and widespread of this disease. The problem to tackle this disease appears to become even worse due to the recent outbreak of SARS-CoV-2. Further, the global number of TB cases are continuously rising which are fueled by poverty, HIV/AIDS, the emergence of multidrug-resistant (MDR) and extremely drug-resistant (XDR) strains of *Mtb*. Additionally, the drug-drug interaction issue with antiretrovirals and antidiabetics is a growing concern. The expanding threat of drug resistance has prompted urgent calls for new approaches to TB control, including the implementation of new modes of drug-susceptibility testing, use of alternative (shorter) therapeutic regimens aimed at expediting diagnosis and treatment, and most importantly to discover compounds (and regimens) with novel mechanisms of action (MoAs) (Singh and Chibale, 2021). The Research Topic aimed to address the current knowledge, research trends, and the future directions of TB drug discovery and development.

The TB drug discovery and development mainly include two approaches for hit identification, the target-based - screening against a particular essential enzyme, and the phenotypic involving screening against wild-type or recombinant whole-*Mtb* cells. In contrast to other infectious disease drug discoveries, in TB, the whole-cell screening followed by elucidation of MoA has been the most successful approach in progressing novel drug-like compounds into the TB drug discovery pipeline (https://www.newtbdrugs.org/pipeline/discovery). Nonetheless, the novel target-based approaches based on new drug targets are coming up and have shown promise (Huszár et al., 2020). In line with this, Oh et al. critically discussed various scaffolds that have been identified in the last 10 years from screens of small molecule libraries against whole-cells or targets where MoA investigation has defined target-hit couples and structure-activity relationship (SAR) studies have described the pharmacophore. The learnings shared by the authors are spot-on, viz. importance of various screening conditions mimicking the host environment; pairing structure-based with whole-cell read-outs was repetitive in yielding discrepancies - often perhaps due to the metabolism of scaffolds by *Mtb* cells; lipophilicity played an important role in whole-cell activity within a scaffold; failure of a series to progress was mainly due to the lack of *in vivo* efficacy in murine models of TB. In the case of phenotypic screening, it is a well-accepted notion in the TB field that deconvoluting the MoA of a phenotypic hit can be challenging and time-consuming. In recent times, considerable efforts have been made to develop a cascade utilizing various biological assays to inform mechanistic information (Mukherjee et al., 2017; Singh and Chibale, 2021). In this context, the study published by Chengalroyen et al. is an important one that describes the application of a set of phenotypic assays to elucidate the MoAs of phenotypic hits.

TB drug discovery pipeline has been satisfactorily busy recently in incorporating new drug-like compounds at various phases of drug development, however, the emergence of resistance to the newly approved drugs such as bedaquiline is concerning. Identification and validation of new drug targets can be a good starting point towards finding a novel drug. Cofactor biosynthetic pathways are established targets for antimicrobial drug development. The review by Butman et al. critically examines the pantothenate and coenzyme A (CoA) biosynthetic enzymes as potential drug targets. They valued that the target assessment of individual CoA biosynthetic enzymes in *Mtb* is not straightforward and should not be reduced to a simple gene essentiality analysis. Their recommendation of combination treatments of drug regimens involving multi-target inhibitors that all have a CoA producing or utilizing enzyme is an interesting one. Similarly, Shaku et al. beautifully reviewed recent developments in identifying cell-wall targets and molecules - critically probing those that specifically inhibit a particular enzyme in cell-wall biosynthesis to those that may indirectly enhance the activity of compounds by weakening the cell-wall.

Finally, the cherry on top for this Research Topic was the beautifully written review on the *in vivo* vertebrate animal models of TB disease used in evaluating compound efficacy. Yang et al. critically review the practical aspects of each model, including the zebrafish, various mice, guinea pigs, rabbits, and non-human primates. This comprehensive review can be considered as a guideline in drawing a rationale for choosing the suitable animal model for progressing the compound.

In summary, this Research Topic relishes the contribution of top-leading scientists aimed at providing a current state of the art knowledge of TB drug discovery and development.

## Author Contributions

The author confirms being the sole contributor of this work and has approved it for publication.

## Conflict of Interest

The author declares that the research was conducted in the absence of any commercial or financial relationships that could be construed as a potential conflict of interest.

## Publisher’s Note

All claims expressed in this article are solely those of the authors and do not necessarily represent those of their affiliated organizations, or those of the publisher, the editors and the reviewers. Any product that may be evaluated in this article, or claim that may be made by its manufacturer, is not guaranteed or endorsed by the publisher.
